# Antiplasmodial activity of iron(II) and ruthenium(II) organometallic complexes against *Plasmodium falciparum* blood parasites

**DOI:** 10.1590/0074-02760150163

**Published:** 2015-12

**Authors:** Nicolli Bellotti de Souza, Anna Caroline Campos Aguiar, Alane Cabral de Oliveira, Siden Top, Pascal Pigeon, Gérard Jaouen, Marilia Oliveira Fonseca Goulart, Antoniana Ursine Krettli

**Affiliations:** 1 Fundação Oswaldo Cruz, Centro de Pesquisas René Rachou, Belo Horizonte, MG, Brasil; 2 Universidade Federal de Alagoas, Instituto de Química e Biotecnologia, Maceió, AL, Brasil; 3 Universidade Federal de Alagoas, Faculdade de Nutrição, Maceió, AL, Brasil; 4 Sorbonne Universités, Université Pierre-et-Marie-Curie, Unités Mixtes de Recherche 8232, Paris, France; 5 Ecole Nationale Supérieure de Chimie de Paris, Paris, France

**Keywords:** Plasmodium falciparum, metallodrug activity, ruthenocifens, ferrocifens

## Abstract

This work reports the in vitro activity against *Plasmodium falciparum*blood forms (W2 clone, chloroquine-resistant) of tamoxifen-based compounds and their ferrocenyl (ferrocifens) and ruthenocenyl (ruthenocifens) derivatives, as well as their cytotoxicity against HepG2 human hepatoma cells. Surprisingly with these series, results indicate that the biological activity of ruthenocifens is better than that of ferrocifens and other tamoxifen-like compounds. The synthesis of a new metal-based compound is also described. It was shown, for the first time, that ruthenocifens are good antiplasmodial prototypes. Further studies will be conducted aiming at a better understanding of their mechanism of action and at obtaining new compounds with better therapeutic profile.

Malaria is estimated to have threatened 198 million people in 2013 (WHO 2014). Resistance of *Plasmodium falciparum* to artemisinin derivatives (Miotto et al. 2013,Ashley et al. 2014) and of *Plasmodium vivax* to chloroquine (CQ) (Graf et al. 2012, Marques et al. 2014) hinders chemotherapy-based efforts to control the disease. *P. falciparum* causes the most deadly form of the disease (WHO 2014), thus new antimalarial drugs are needed, especially towards CQ-resistant parasites.

The potentiality of the metal-based approach to discover new drugs has been highlighted by ferroquine, which proceeded to Phase IIB clinical trials as an antimalarial drug (Biot 2004, Biot et al. 2012a, Held et al. 2015b). Very recently, the combination of ferroquine with artesunate was shown to be safe at all doses tested, associated with high cure rates. Therefore it represents a promising alternative for drug combination against *P. falciparum*malaria (Held et al. 2015b). Ferroquine is the only candidate in Phase II clinical trials that has a half-life longer than 20 days, allowing for a prolonged post-treatment prophylactic effect and diversifying the antimalarial portfolio (Held et al. 2015a). Experimentally, two other ferrocene derivatives have shown important antiplasmodial activity (Soares et al. 2010).

The ruthenium (Ru)-based compounds also attract interest due to their biological activities as anticancer (Pizarro et al. 2010), antibacterial (Wenzel et al. 2013), leishmanicidal, trypanosomicidal (Martínez et al. 2012), antiplasmodial (Biot et al. 2007, Glans et al. 2012), including Ru-CQ complexes (Martínez et al. 2009, Rajapakse et al. 2009). Ruthenocenyl compounds were also described as bioprobes of ferroquine, used in an attempt to elucidate its molecular mechanism of action (Biot et al. 2012b). The use of Ru allowed to evercome the difficulty of detecting iron (Fe)-based compounds among the numerous Fe-containing components of the parasite digestive vacuole (DV) (Dubar et al. 2011, 2012).

An enhanced antiplasmodial activity has been obtained by complexation with Ru in relation to the free ligands, providing molecules such as Ru-lapachol complexes (Barbosa et al. 2014) and Ru-pyridil ester (Chellan et al. 2014), or ether complexes (Chellan et al. 2013), as well as thiosemicarbazone Ru-arene complexes (Adams et al. 2013). Another example of successful complexation of Ru with an antifungal agent (clotrimazole) has led to antiparasitic compounds over 50-fold more potent in relation to the parental compounds (Martínez et al. 2012). Furthermore, the substitution of Fe by Ru in ferroquine led to higher anti-*P. falciparum* activity against K1 strain, another resistant parasite strain (Beagley et al. 2003).

Several ferrocenyl derivatives of tamoxifen demonstrate antiproliferative activity against breast cancer cells (Tan et al. 2012,Cázares-Marinero et al. 2014, de Oliveira et al. 2014).

The present paper reports the evaluation of tamoxifen-based compounds and their ferrocene and ruthenocene derivatives, designed as ferrocifens and ruthenocifens for: (i) antiplasmodial activity against *P. falciparum* (W2 clone, CQ-resistant) blood parasites in culture, and (ii) cytotoxicity in vitro against HepG2 human hepatoma cells. This is the first report dealing with ruthenocifens as antiplasmodial compounds. The synthesis of a new ferrocenophane is also described.

## MATERIALS AND METHODS

Compounds 1, 2, 3, 4, 5, 6, 7, 9, 10, 11, 12, and 13 were prepared according to literature procedures (references are given in Table I). The synthesis of compounds 8 is described in the present paper. Tetrahydrofuran (THF) was distilled over sodium/benzophenone prior to use. Thin layer chromatography was performed on silica gel 60 GF_254_. ^1^H and ^13^C-NMR spectra were acquired on a Bruker 300 MHz spectrometer. Mass spectrometry was carried out at the Mass Spectrometry Service at National Chemical Engineering Institute, Paris. High resolution mass spectra (HRMS) were acquired in the Paris Institute of Molecular Chemistry (Mixed Research Unit 8232) at the Pierre and Marie Curie University, Paris.

**TABLE I:**
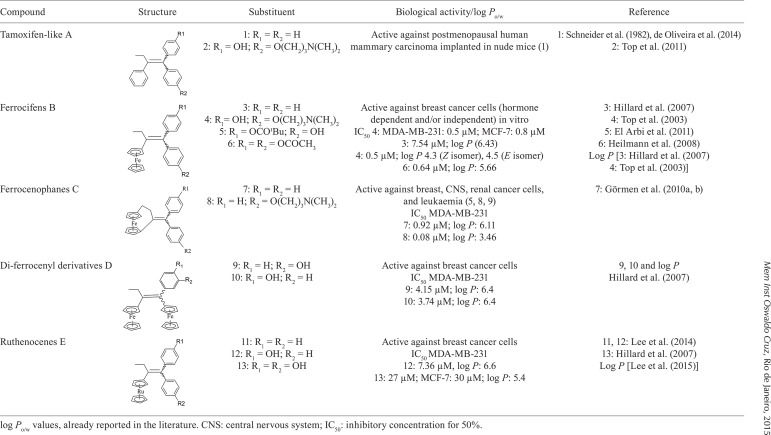
IC50 values of metallocifens against breast cancer cell lines (hormone-independent MDA-MB-231 and hormone-dependent MCF-7)

**Figure:**
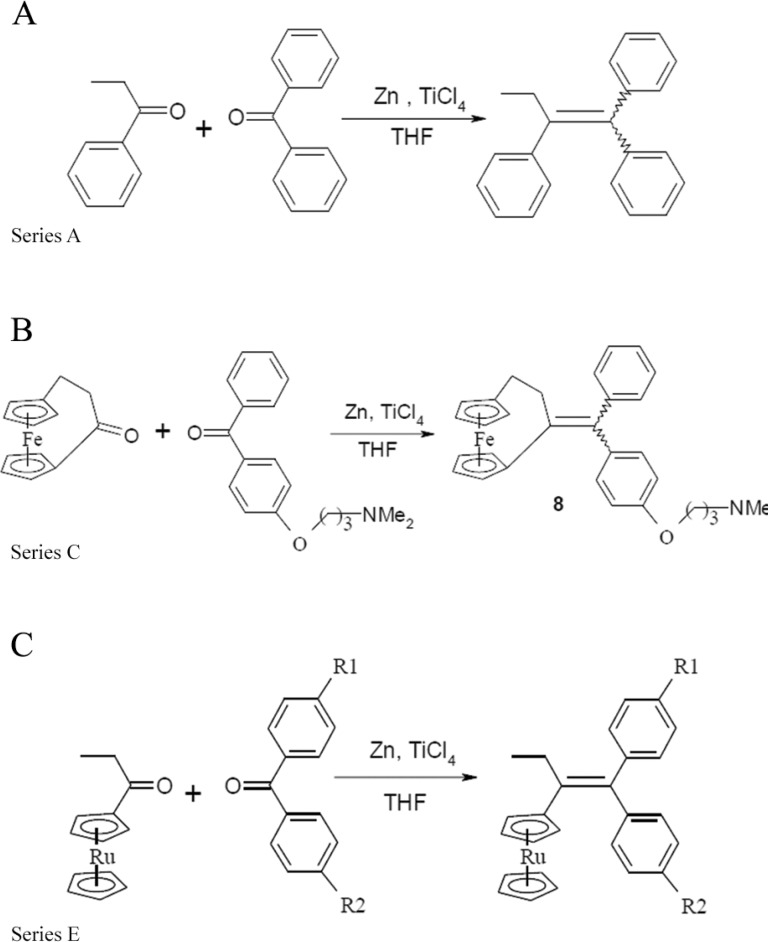
General scheme for the synthesis of compounds of series A (A), C (B), represented by the synthesis of the new compound 8, and E (C). The same procedure was used for the other series with adequate precursors.

*Measurement of lipophilicity data* - Measurements of the octanol/water partition coefficient (log *P*_o/w_) were made by the HPLC technique according to a method described previously (Minick et al. 1988, Pomper et al. 1990). Measurement of the chromatographic capacity factors (*k*) for each molecule was done at various concentrations in the range of 95-75% methanol containing 0.25% (v/v) 1-octanol, and an aqueous phase consisting of 0.15% (v/v) n-decylamine in the buffering agent 3-morpholinopropane-1-sulfonic acid (MOPS) prepared in 1-octanol saturated water adjusted to pH 7.4. These capacity factors (*k’*) are extrapolated to 100% of the aqueous component given the value of *k’*. The log*P*_o/w_ is obtained by the formula log*P*_o/w_ = 0.13418 + 0.98452 log*k*’.

*Synthesis of 1-[(4-(3-dimethylaminopropoxy)phenyl-phenyl)methylidene][3]ferrocenophane, 8* - Titanium chloride (10.04 g, 5.8 mL, 52.9 mmol) was added dropwise to a suspension of zinc powder (4.84 g, 74 mmol) in dry THF (400 mL) at 10-20ºC. The mixture was heated at reflux for 2 h. A second solution was prepared by dissolving [3]ferrocenophan-1-one (2.54 g, 10.6 mmol) and 4-(3-dimethylaminopropoxy)benzophenone (3 g, 10.6 mmol) in dry THF (25 mL). This latter solution was added, dropwise, to the first solution and then the reflux was continued for 4 h. After cooling to room temperature, the mixture was stirred with water and dichloromethane. The mixture was acidified with diluted hydrochloric acid until dark colour disappeared, then, sodium hydrogenocarbonate was added to maintain a pH close to neutral and the mixture was decanted. The aqueous layer was extracted with dichloromethane and the combination of organic layers was dried on magnesium sulphate. After concentration under reduced pressure, the crude product was chromatographed on silica gel column with acetone as the eluent, then was purified by semi-preparative HPLC [Shimadzu apparatus with a Nucleodur C18 column (l = 25 cm, 1 = 3.2 cm, particle size = 10 mm] with a solution of methanol/triethylamine 95/5, as the eluent, giving an undetermined 2/1 ratio of *Z* and*E* isomers. Compound 8 (yield of 84%) was re-crystallised from diethyl ether and was obtained as a bright yellow product as an undetermined 4/1 ratio of *E* and *Z* isomers. ^1^H NMR (CDCl_3_, 300 MHz): δ 1.82-2.04 (m, 2H, CH_2_), 2.23 and 2.27 (s, 6H, NMe_2_), 2.31-2.53 (m, 4H, CH_2_N+CH_2_ cycle), 2.60-2.68 and 2.68-2.75 (m, 2H, CH_2_ cycle), 3.90 (t, *J* = 6.4 Hz, 2H, CH_2_O major isomer), 3.94-4.07 (m, 10H, CH_2_O minor isomer+C_5_H_4_ major and minor isomers), 4.21 (t,*J* = 1.8 Hz, 2H, C_5_H_4_ major isomer), 6.61 and 6.88 (d, *J* = 8.8 Hz, 2H, C_6_H_4_), 6.94 and 7.14 (d, *J* = 8.8 Hz, 2H, C_6_H_4_), 7.02-7.10 (m, 1H, C_6_H_5_), 7.20-7.39 (m, 4H, C_6_H_5_).^13^C NMR (CDCl_3_, 75.4 MHz): δ 27.4 and 27.5 (CH_2_), 28.7 (CH_2_), 40.9 (CH_2_), 45.3 (2CH_3_ NMe_2_), 56.4 (CH_2_), 65.9 and 66.1 (CH_2_O), 68.2 (2CH C_5_H_4_), 68.5 and 68.7 (2CH C_5_H_4_), 70.2 (2CH C_5_H_4_), 70.3 (2CH C_5_H_4_), 83.7 (C_ip_), 86.7 and 86.8 (C_ip_), 113.2 and 114.0 (2CH C_6_H_4_), 125.9 and 126.6 (CH C_6_H_5_), 127.2 and 128.1 (2CH_arom_), 129.3 and 130.4 (2CH_arom_), 130.6 and 131.6 (2CH_arom_), 133.6 and 134.3 (C), 135.5 and 135.9 (C), 140.5 and 140.6 (C), 143.4 and 143.8 (C), 157.1 and 157.7 (C). MS (EI, 70 eV) *m*/*z*: 491 [M]^+^, 405 [M-NMe_2_CH_2_CH_2_]^+^, 86 [NMe_2_CH_2_CH_2_]^+^, 58 [NMe_2_CH_2_]^+^. HRMS (ESI, C_31_H_34_FeNO: [M+H]^+^) calculated: 492.1990, found: 492.1998.

*Cytotoxicity tests with HepG2 human hepatoma cells and monkey kidney (BGM) cell lines* - Cytotoxicity tests were performed with HepG2 human hepatoma cells or normal BGM cell lines using 3-(4,5-dimethylthiazol-2-yl)-2,5 diphenyltetrazolium bromide (Molecular Probes, USA) (Denizot & Lang 1986) or neutral red (Borenfreund et al. 1987) methods. The minimum lethal dose for 50% of the cells (MLD_50_) was determined (de Madureira et al. 2002) by a curve-fitting software (Microcal Origin Software v.5.0; Origin Lab Co, USA) and further used to calculate the selectivity index (SI) of the active compounds [SI = MDL_50_/inhibitory concentration for 50% (IC_50_)] (Bézivin et al. 2003). The SI was calculated in order to give an insight into the therapeutic index of the molecules, i.e., how far the toxic concentration is from the therapeutic one. Molecules having MLD_50_ > 500 mM were considered not toxic, if between 500-100 mM moderately toxic, and those having MLD_50_< 100 mM were considered toxic. Molecules with SI ≤ 10 were also considered toxic.

*Continuous culture of P. falciparum and in vitro tests of drug activity* - Blood-stage *P. falciparum* parasites, W2 clone CQ-resistant (Oduola et al. 1988), maintained according to Trager & Jensen (1976), were used in the drug activity tests after sorbitol-synchronisation (Lambros & Vanderberg 1979). The antiplasmodial activity of the compounds was determined relative to control parasites kept in culture medium only (Rieckmann et al. 1978) through the anti-histidine-rich protein II assay (Noedl et al. 2002). The IC_50_ of parasite growth was determined through sigmoidal dose-response curves built by curve-fitting software (Microcal Origin Software v.5.0). Compounds exhibiting IC_50_ values lower than 6 mM were considered active, those with IC_50_ between 20-60 mM partially active, and those higher than 60 mM, inactive.

## RESULTS

The compounds evaluated in this work belong to five structural classes and their reported biological activities and some physicochemical parameters are listed inTable I. They are classified as: organic tamoxifen-like compounds in series A (1, 2), compounds having a ferrocenyl substituent in series B (3-6), a [3]ferrocenophane substituent (7, 8) in series C, two ferrocenyl groups (9, 10) in series D, and the ruthenocenes (11-13) in series E. Substitutions on the phenyl rings include hydroxyl, acetoxy, pivaloxy, and/or dimethylaminopropoxy groups.

Log *P* values were determined for the first time in this paper, for compounds 6, 7, and 8, being: 5.66, 6.11, and 3.46, respectively. All log*P* values are higher than 4 (except for compound 8, whose log*P* was 3.46), pointing to a lipophilic trend of the compounds. They can be ranked in a general fashion as follows (in decreasing order): diferrocenyl derivatives > ruthenocenes > tamoxifen-like derivatives > ferrocifens, intermixed with the more lipophilic compound 3 (log *P*= 6.43), and the more hydrophilic one, compound 8 (log *P* = 3.46).

Most of the compounds were synthesised using a McMurry cross-coupling reaction (1, 3, 7, 8, 9, 10, 11, 12, 13), or by functionalisation of a phenolic compound that were synthesised by this method (2, 4, 5, and 6) (Figure). Synthesis had been performed as previously reported for all compound classes summarised in Table I, except for compound 8, prepared using a McMurry reaction between the [3]ferrocenophan-1-one (Turbitt & Watts 1972), and the 4-(3-dimethylaminopropoxy)benzophenone (Top et al. 2002), yielding 84%.

Among the 13 tested compounds, six were active against *P. falciparum*CQ-resistant parasites based on the IC_50_ values (Table II). The most active compounds (2 and 4) showed IC_50_ below 6, followed by compounds 8, 12, 13, with IC_50_values below 6 mM; compounds 3 and 11 were partially active (IC_50_values around 16.6 mM), and compounds 1 and 7, with IC_50_ values above 60 mM, were considered inactive. These results show a special effect of the dimethylaminopropoxy chain, since the compounds bearing it (2, 4, and 8) ranked the first three places of activity.

**TABLE II:** Selectivity indexes (SI), the ratio between in vitro cytotoxicity [minimum lethal dose for 50% of the cells (MLD50)] and activity [inhibitory concentration for 50% (IC50), mM] against *Plasmodium falciparum* (Pf) of tamoxifen-like compounds and metallic derivatives

Compounds/	Structural	MLD_50_	IC_50_	SI
series	class	HepG2^*a*^	Pf	(MLD_50_/IC_50_)
1/A	Tamoxifen-like	> 3516	83 ± 5	42
2/A		< 10	2.2 ± 0.05	Toxic
3/B	Ferrocifene	479 ± 89	16.6 ± 2.3	29
4/B		< 7.7	0.7 ± 0.1	Toxic
5/B		< 61	23.6 ± 9.8	Toxic
6/B		< 61	23.6 ± 5.9	Toxic
7/C	[3]ferrocenophane	> 2562	62.8 ± 10.7	41
8/C		< 63	5.9 ± 1.6	18
9/D	Di-ferrocenyl derivative	< 60	27.1 ± 23.2	Toxic
10/D		< 60	7.8 ± 1.6	Toxic
11/E	Ruthenocene	2248 ± 53	16.5 ± 0.5	136
12/E		251 ± 34	4.7 ± 1.3	53
13/E		266 ± 3	5.9 ± 2.3	45
CQ	Quinoline	502 ± 52	0.1 ± 0.02	5,020

*a*: except for compounds 12 and 13, which were tested for cytotoxicity against normal monkey kidney cells using the neutral red method; CQ: chloroquine.

Regarding the in vitro cytotoxicity tests against HepG2 cells, compounds 1, 7, and 11 exhibited MLD_50_ value up to 3,516 mM, compounds 3, 12, and 13, MLD_50_values ranging from 479-266.2 ± 3 mM, being considered nontoxic and moderately toxic, respectively. Remaining compounds (2, 4, 5, 6, 8, 9, and 10) were considered toxic (MLD_50_ values below 100 mM), especially compounds 2 and 4, with MLD_50_ values below 10 mM.

The compounds were ranked in relation to their SI (Table II, column 5) as: 11 > 12 > 13 > 1 > 7 > 3. The other compounds exhibited low SI due to their high toxicity towards HepG2 cells.

## DISCUSSION

Based on the present and published data (Soares et al. 2010), some interesting trends emerge. The presence of the dimethylaminopropoxy side-chain increases antiplasmodial activity, with IC_50_values to around 2.2 ± 0.05 mM (for 2) and 0.7 ± 0.1 mM (for 4), and also their cytotoxicity, in comparison to 1 and 3, respectively. In addition, we have shown that hydroxy moieties in *para* position, or biologically hydrolysable ester groups, as in 6 (Heilmann et al. 2008, Görmen et al. 2010a), also increase the cytotoxicity (Hillard et al. 2007). For this reason, compound 8 bearing only a dimethylaminopropoxy chain has lower cytotoxicity than 2 and 4. Compounds having no substituent on the phenyl moieties had the lowest activities on HepG2 cells (1, 7, 3, and 11). The presence of the ferrocenyl group increases more than three times the antiplasmodial activity (1 vs. 3, and 2 vs. 4) (Table II). The toxicity also increased, thus diminishing the SI to undesirable values, as observed previously with cancer cell lines (de Oliveira et al. 2011). The compounds 4, 6, 9, and 10 become too toxic for *P. falciparum*. By contrast, ferrocenophane compounds 7 and 8 appear to be less toxic (SI = 41 and 18, respectively).

Interestingly, SI of ruthenocene compounds are better than that of ferrocene compounds. The IC_50_ value for 11 (16.5 ± 0.5 mM) is similar to that of 3 (16.6 ± 2.3 mM). By contrast, MLD_50_ values for these two compounds are very different, 2248 ± 53 mM vs. 479 ± 89 mM. The presence of a phenol moiety in the ruthenocifen series increases not only the antiplasmodial, but also the cytotoxic activity (compound 12 and 13). Compound 11 appears to have the best profile, with SI > 100. Low cytotoxicity of ruthenocenyl compounds, as compared to ferrocenyl compounds, was also observed for breast cancer cells (Gobec et al. 2014, Lee et al. 2015). Concerning different activities between ferrocenyl and ruthenocenyl compounds, it may well be due to their selective cytotoxicity. A recent work dealing with some of the molecules presented herein (Lee et al. 2015) attributed this differential cytotoxicity to the solubility and stability of the quinone-methide (QM) moieties formed after oxidation, as well as the rapidity of this process (ferrocenes form QM faster than ruthenocenes, whose phenoxy radicals are not turned into QM moieties rapidly). The nature of the metallocene, which include redox properties and acidity of the phenolic proton of the radical cations also play a role. Ruthenocenic derivatives of peptide nucleic acids were also shown to be less toxic than the ferrocenic ones, which can be due to the higher chemical and oxidative stability of ruthenocene, in relation to ferrocene (Swarts et al. 2009).

Despite the use of few compounds for comparison in this work (1 vs. 3 vs. 11; 2 vs. 4) and the absence of mechanistic studies, due to the extreme complexity of inherent possible events related to metal complexes (Gasser et al. 2011, Coogan et al. 2012), it is possible to suggest that the presence of redox-active metal centres increases the biological activity. Drug lipophilicity facilitates membrane permeability, providing accumulation of drug in the resistant parasite DV. This is possibly the cause for the increase of efficacy of organometallic compounds (Martínez et al. 2009, Rajapakse et al. 2009, Dubar et al. 2011, 2012, Glans et al. 2012).

In fact, log *P* values reported for the metallocenes presented herein suggest that these molecules can cross cell membranes readily. Within each series, there is no significant difference among the two metals (Ru, Fe) and the lipophilicity decreases in the order monophenol > diphenol > tamoxifen-like compounds. This is the trend expected for the addition of an hydroxyl group or an amino chain, the latter responsible for a stronger decrease (Lee et al. 2015).

Concerning specifically the structural classes of the present studied compounds toward cancer cells, electrochemical and biochemical studies (Pigeon et al. 2005, Hillard et al. 2007, Nguyen et al. 2007) pointed to the involvement of oxidative formation of cytotoxic quinone-type metabolites in the activity of many ferrocifens, inactivating proteins, or increasing oxidative stress in cells, leading to cells death (Nguyen et al. 2007, Hamels et al. 2009, Lee et al. 2014, 2015). Thus, the generation of reactive oxygen species may represent a mode of action against *P. falciparum*, as observed for other tamoxifen-like molecules bearing ferrocene moiety (Soares et al. 2010).

Some Ru complexes were shown to be kinase inhibitors (Debreczeni et al. 2006). They also inhibited thioredoxin reductase (Casini et al. 2008) which is an important system responsible for redox homeostasis in *P. falciparum*(Kanzok et al. 2000). Indeed, a recent study showed that ferrocenyl derivatives of tamoxifen, including some of those studied herein, targeted thioredoxin reductases of cancer cells (Citta et al. 2014).

Falcipain-2, a cystein protease involved in haemoglobin degradation in *P. falciparum* (Chugh et al. 2013), is also a likely target for compound 11, since cystein proteases are amenable to be attacked by metals (Fricker 2010).

Other potential targets for ruthenocenic compounds include DNA and parasite proteins (Gambino & Otero 2012), as shown for tumour cells (Brabec & Nováková 2006,Casini et al. 2008). Indeed, Ru-arene complexes have been designed to interact with DNA by intercalation and methylation (Aird et al. 2002). Despite no DNA-interaction studies were performed with the ruthenocifens presented herein, a recent report (de Oliveira et al. 2014)proved, by performing differential pulse voltammetry and spectrophotometric analysis, the interaction of compound 3 with double-stranded DNA and single-stranded DNA.

In conclusion, along with describing the synthesis of a new ferrocenophane, this work represents an additional evidence for the metal-complex approach enhancing the antiplasmodial activity, with emphasis to ruthenocifens, for the first time assayed against resistant *P. falciparum* parasites, showing the best therapeutic potential. Several possible modes of action are discussed, by comparison with the literature. A further structural optimisation is required in order to evaluate a larger library of such compounds, which is under way, together with investigation of the mechanism of action, based on the bioprobe potential use of Ru derivatives.
